# Molecularly Mixed Composite Membranes for Gas Separation Based on Macrocycles Embedded in a Polyimide

**DOI:** 10.3390/polym16040460

**Published:** 2024-02-07

**Authors:** Danilo Vuono, Gabriele Clarizia, Loredana Ferreri, Grazia Maria Letizia Consoli, Daniela Clotilde Zampino, Giuseppina Scalzo, Salvatore Petralia, Paola Bernardo

**Affiliations:** 1 Institute on Membrane Technology (ITM-CNR), 87036 Rende, Italy; 2 Institute of Biomolecular Chemistry (ICB-CNR), 95126 Catania, Italy; 3 Institute of Polymers, Composites and Biomaterials (IPCB-CNR), 95126 Catania, Italy; 4 Department of Drug and Health Sciences, University of Catania, Via Santa Sofia 64, 95125 Catania, Italy

**Keywords:** calixarenes, polymer membranes, gas separation, transport properties

## Abstract

Polyimides are a polymer class that has been extensively investigated as a membrane material for gas separation owing to its interesting permselective properties in a wide range of operation temperatures and pressures. In order to improve their properties, the addition of different filler types is currently studied. *p-tert*-Butylcalix[n]arene macrocycles (PTBCs) with different cavity sizes (PTBC4, PTBC6, PTBC8) were used as fillers in a commercial thermoplastic polyimide, with a concentration in the range 1–9 wt%, to develop nanocomposite membranes for gas separation. The selected macrocycles are attractive organic compounds owing to their porous structure and affinity with organic polymers. The nanocomposite membranes were prepared in the form of films in which the polymeric matrix is a continuous phase incorporating the dispersed additives. The preparation was carried out according to a pre-mixing approach in a mutual solvent, and the solution casting was followed by a controlled solvent evaporation. The films were characterized by investigating their miscibility, morphology, thermal and spectral properties. The gas transport through these films was examined as a function of the temperature and also time. The results evidenced that the incorporation of the chosen nanoporous fillers can be exploited to enhance molecular transport, offering additional pathways and promoting rearrangements of the polymeric chains.

## 1. Introduction

Membrane gas separation is a well-established technique with attractive features compared to conventional energy-intensive separation processes such as cryogenic distillation or adsorption [1,2]. Aromatic polyimides are among the few polymeric materials industrially used to produce membranes for gas separation [1]. Due to their chemical and mechanical resistance, polyimides are an interesting starting point for the preparation of nanocomposite membranes via the addition of selected additives. Different studies investigated the loading of solid particles within a polyimide matrix, producing Mixed Matrix Membranes (MMMs) having enhanced separation performances compared to the neat polymeric membranes [3,4,5]. Fillers with an intrinsic porosity could be able to enhance the permeation flux through the membranes. Affinity issues limit the filler amount of inorganic particles, leading to aggregates and defective assemblies. Instead, an organic structure or the presence of organic moieties in the fillers can help the compatibility of the phases that have a common nature, resulting in a better polymer/filler interaction.

In this view, macrocycles are porous organic fillers that can be advantageously included within polymeric membranes in order to obtain advanced MMMs [6]. Among macrocycles, the calix[n]arene family offers vase-like oligomers formed by a number *n* of phenolic units linked by methylene bridges that define a hydrophobic cavity with a distinctive 3D structure and a variable size depending on the number of phenolic units forming the macrocycle [7]. The presence of a cavity capable of complexing ions and neutral molecules, including biomolecules [8] or drugs [9,10], makes the calix[n]arenes the third generation family of supramolecular hosts, after crown ethers and cyclodextrins. Calix[4–8]arenes made of four to eight arene units are the most studied oligomers. Compared to cyclodextrins, calix[n]arenes are flexible and not soluble in water, and they offer the advantage of a remarkable synthetic versatility [11]. A variety of functional groups, similar to or different from each other, can be introduced in the calix[n]arene lower and upper rim, which are formed by the phenol OH groups and aromatic rings, respectively [12,13]. The opportune functionalization of the calix[n]arene lower and upper rim permits to control the macrocycle conformational mobility and address and enforce the host properties, depending on the cavity size and shape [14], and on the additional recognition sites introduced in the macrocycle [15]. Thus, it is possible to take advantage of the intrinsic porosity of calixarenes by introducing additional permeation paths to a polymeric matrix, enhancing membrane permeability. On the other hand, their recognition ability can potentially boost the membrane selectivity. In addition, compared to purely inorganic fillers, the organic molecular structure of the selected additives helps the compatibility of the macrocycles with the polymeric matrix. Therefore, calixarenes are highly promising for the development of next-generation membranes, as recently evidenced by Chung and Lai [16].

The use of calixarenes entrapped within polymeric membranes for gas separation was reported in a few studies [17,18]. Calix[4]arene and calix[8]arene derivatives were loaded into a highly permeable polymer (i.e., 3-trimethylsilyltricyclononene-7, PTCNSi1), in the 9–29 wt% concentration range [17]. Compared to the neat polymer, the hybrid membranes presented a reduced permeability and an enhanced selectivity. In particular, the membranes prepared with larger size calixarenes were to some extent closer to the Robeson upper bounds for He/N_2_ and H_2_/CH_4_ gas pairs.

Instead, a high compatibility of the *p-tert*-butylCalix[4]arene with a polyether block amide (Pebax 1657) was reported by Nadeali et al. that prepared membranes for CO_2_ separation [18]. The Calix[4]arene displayed an even distribution in the polymer and significantly increased the CO_2_ flux and the CO_2_ selectivity (CO_2_/N_2_ and CO_2_/CH_4_). The optimum Calix[4]arene loading was 0.75 wt% with an enhancement of 88% for the CO_2_ permeability (from 141 to 265 Barrer) with respect to the neat Pebax 1657 membrane. The CO_2_/CH_4_ selectivity has been improved by ca. 100% (from 25 to 51) and by 150% (from 43 to 109) for CO_2_/N_2_. The enhanced selectivity for CO_2_ separation was attributed to the interactions between the carbon atom of CO_2_ with the oxygen atom in the hydroxyls of the calix[4] arene as well as its interaction with the benzene rings.

A systematic study showed that the tert-butyl functional group in the para position of the calix[4] arene aromatic rings leads to calixarenes being among the top performing in terms of affinity with CO_2_ [19]. *p-tert-*butylcalix[4] arene selectively adsorbs components O_2_ and CO_2_ from air. Cooperative rotation of the *tert-butyl* groups about the C(Ar)–C(sp^3^) bond help the diffusion of small gas molecules through the molecular crystals without disturbing the arrangement of the macrocyles [20].

A simulation study of membranes based on a commercial aromatic polyimide (Matrimid^®^) filled with Calix[4]arene (0–1.5%) predicted improved CO_2_ permeability, CO_2_/N_2_ and CO_2_/CH_4_ selectivity compared to the neat polymeric membrane with a maximum at a concentration of 0.75 vol% of calixarene [21].

Based on the promising evidence, in the present work, the potential of these original fillers was combined with the well-established transport properties of a commercial polyimide taking advantage of their common organic nature in an appropriate concentration range, suitable for scaling on an industrial level.

For the first time, three *p-tert*-butylcalix[4,6,8] arenes with a different cavity size were included within Matrimid^®^, producing self-supported membranes suitable for gas separation. 

Blending was adopted for the membrane manufacturing since it is a straightforward method with no additional steps and, thus, it can be applied on large-scale production. The explored concentration range of the organic additives in the membranes was up to 9 wt%. The membranes were characterized, investigating their structural features, thermal behavior and gas transport properties.

## 2. Experimental

### 2.1. Materials

**Figure 1:**
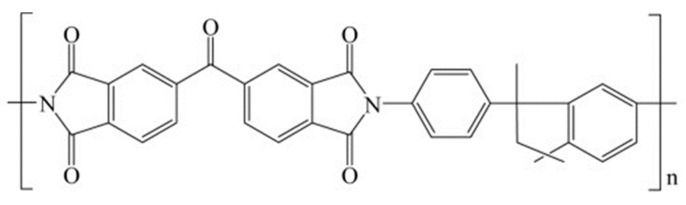
Chemical structure of Matrimid^®^5218.

The polyimide Matrimid^®^5218 (3,3′,4,4′-benzophenonetetracarboxylic dianhydride and diaminophenylindane), having the chemical structure shown in Figure 1, was provided by Huntsman Advanced Materials American (USA). The bulk density of Matrimid^®^ is 1.24 g cm^−3^.

**Figure 2:**
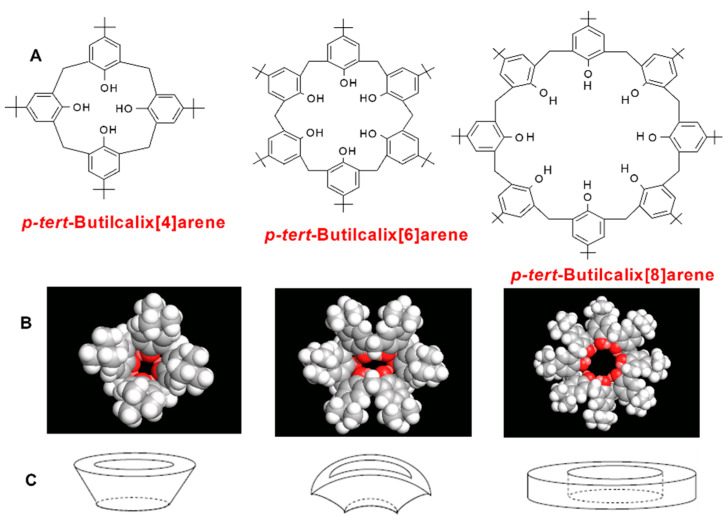
*p-tert*-Butylcalix[4,6,8]arenes: (**A**) chemical structure; (**B**) molecular modeling images (*cone*, *pinched-cone* and *pleated-loop* conformation); (**C**) schematic representation of the calixarene cavity shape.

The calix[4,6,8]arenes, purchased from Sigma Aldrich (Milan, Italy), present *p*-*tert*-butyl groups on the upper rim and OH groups on the lower rim (Figure 2). They differ for the ring size and, thus, for the molar volume (Table 1).

Dichloromethane (DCM) was purchased from VWR International (Milan, Italy) and utilized as a solvent for membrane preparation. The chemicals were used as received.

Gases for permeation tests (H_2_, He, CO_2_, O_2_, N_2_ and CH_4_) were purchased by SAPIO (Monza, Italy) with a purity of 99.99%.

### 2.2. Methods

#### 2.2.1. Membrane Preparation 

The polymeric solution was prepared by dissolving the polyimide at a concentration of 2 wt% in dichloromethane (DCM). 

Weighted amounts of the additives were introduced into the polymeric solution. DCM was selected to prepare the polymeric solution since it can ensure also the calixarene solubility. The solution was left under stirring for a few hours. Indeed, a quick polymer dissolution was observed in DCM, under stirring at room temperature, also in the presence of the calixarenes at 9 wt%. 

Dense nanocomposite films, referred to as M/PTBC, were obtained according to a controlled solvent evaporation procedure, pouring fixed amounts of the dope solution within a stainless-steel ring placed on a perfectly flat glass plate. Neat polymer samples were prepared as well for comparison.

#### 2.2.2. Thermogravimetric Analysis (TGA) 

The thermogravimetric analysis was performed on the PTBCs and on the prepared membranes from 40 to 800 °C under a nitrogen atmosphere at a heating rate of 10 °C/min. The weight loss percentage and its derivative (DTG) were recorded as a function of temperature using a TGA apparatus (TA Instruments Q500, Milan, Italy) on samples of ca. 4–5 mg. 

#### 2.2.3. Calorimetric Measurements (DSC)

Calorimetric measurements were carried out on the calixarenes and on the membranes using a Differential Scanning Calorimeter (DSC) equipped with a sub-ambient accessory (TA Instruments Q100, Milan, Italy). The instrument was calibrated with high-purity standards (indium and cyclohexane) using nitrogen as a purge gas. An empty aluminum pan was used as reference. The weight of the analyzed samples was in the range 3–5 mg. Heating and cooling cycles were performed from −90 to 350 °C at a rate of 10 °C/min. Two heating cycles were carried out: the first of which to erase the previous thermal history. 

#### 2.2.4. Fourier Transform Infrared Spectroscopy–Attenuated Total Reflection (FTIR-ATR) Analyses

The FTIR-ATR measurements were recorded on the calixarenes and on the films using the FT-IR Imaging System Frontier Spotlight 400 (Perkin Elmer, Milan, Italy) in the range 4000–550 cm^–1^ with 4 cm^–1^ resolution and 16 scans. Atmospheric corrections were applied to the background. In the case of the neat calixarenes, the powders were placed on the ATR crystal by applying a pressure to be adhered completely until the signals became stable.

#### 2.2.5. Nuclear Magnetic Resonance Spectra (NMR) 

The M/PTBC8 9% film (11 mg), PTC8 (1 mg) and Matrimid^®^5218 (10 mg) powders were dissolved in CDCl_3_ solvent, and ^1^H NMR spectra (400.13 MHz) were acquired on a Bruker Avance 400 spectrometer (Billerica, MA, USA) at 297 K.

#### 2.2.6. Scanning Electron Microscopy (SEM)

A Thermo Phenom Prox desktop SEM (Thermo Fisher Scientific, Waltham, MA, USA) equipped with an integrated energy-dispersive X-ray (EDX) detector was used to analyze the morphology of neat Matrimid and M/PTBC films. Samples, prepared by fracturing them in liquid nitrogen, were dried and sputter-coated with gold. Micrographs were acquired at 15 KV. 

#### 2.2.7. Gas Permeation Tests on the Membranes

Gas permeation tests with single gases (H_2_, He, N_2_, O_2_, CO_2_ and CH_4_) were carried out at 25 °C on “fresh” flat dense membranes having an effective area in the range of 2.14–11.3 cm^2^. The experiments were further performed on selected samples varying the temperature and tracking the permeation during time to investigate their behavior upon aging. A fixed volume/pressure increase instrument (Elektro & Elektronik Service Reuter, Geesthacht, Germany) [22] were used. Before each experiment, the film sample was deeply evacuated with a turbomolecular pump included in the set-up in order to ensure the careful removal of previously dissolved species. The membrane was put in contact with the test gas at pressure values up to 1 bar, tracking the increase over time of the permeate pressure in a calibrated volume. The gas permeability (*P*) was calculated from the slope of the linear part of the pressure curve at steady-state condition:

(1) $$P=\frac{T_{0}}{T}\cdot \frac{1}{p_{0}}\cdot \frac{l\cdot V_{P}}{A\cdot p_{feed}}\cdot \frac{dp}{dt}$$

where *T*_0_ and *p*_0_ are the standard temperature and pressure (i.e., 273.15 K and 76 cmHg), T is the operating temperature (K), *l* is the thickness of the membrane (cm), *V_P_* is the volume of the permeate chamber (cm^3^), *A* is the active membrane area (cm^2^), *p_feed_* is the pressure of the feed gas (cmHg), and *dp/dt* is the slope of the increasing rate of pressure in the permeating volume in the linear region (cmHg s^−1^), and P is expressed in Barrer units (1 Barrer = 10^−10^ cm^3^ (*STP*) cm cm^−2^ s^−1^ cmHg^−1^ = 3.35 10^−16^ mol m^−2^ s^−1^ Pa^−1^).

The gas time lag (*θ*) was obtained by extrapolating the linear section of the curve on the abscissa. Thus, the diffusion coefficient, *D*, of each gas through the membrane can be evaluated as [23]:

(2) $$D=\frac{l^{2}}{6\theta }$$

The ideal selectivity was calculated by dividing the individual permeability values for two gases A and B:

*α*_A/B_ = *P*_A_/*P*_B_ (3)

A schematic diagram of the permeation testing unit is provided in Figure S1.

The membrane thickness was measured using a digital micrometer (Mitutoyo 543–561D Metric Dial Indicator, Milan, Italy) and averaging multiple point measurements for each sample.

## 3. Results

The M/PTBC membranes were prepared by a pre-mixing approach, according to a solution-casting technique with controlled solvent evaporation, and characterized for miscibility, morphology, thermal and spectral properties, and gas transport performance.

### 3.1. TGA

**Figure 3:**
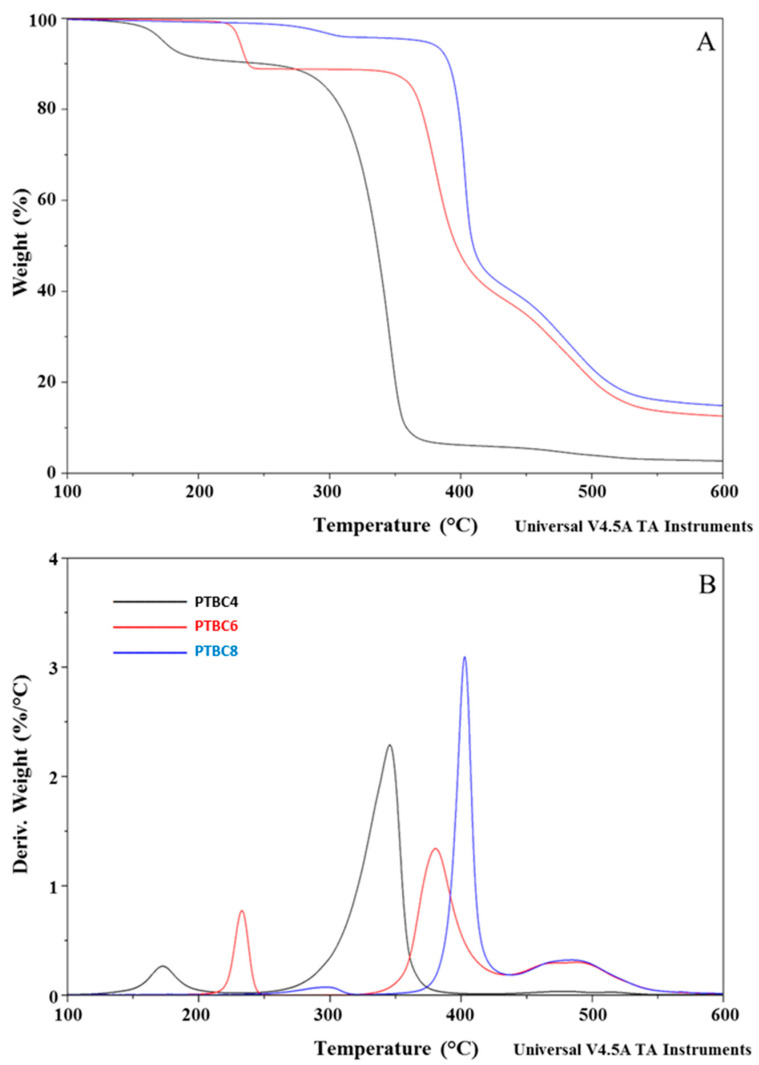
TGA (**A**) and DTG (**B**) curves overlay of neat PTBC4, PTBC6 and PTBC8. The legend refers to both graphs.

**Figure 4:**
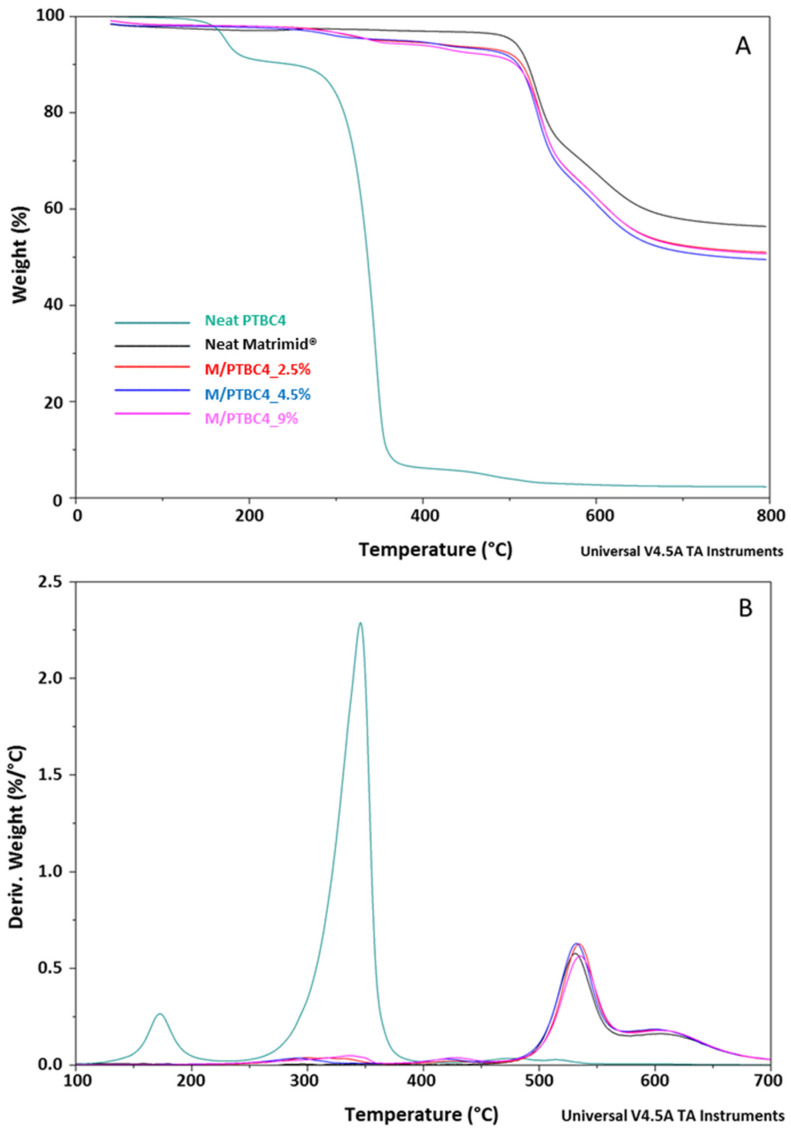
TGA (**A**) and DTG (**B**) curves of the PTBC4, neat Matrimid^®^ and Matrimid^®^/PTBC4 blends. The legend refers to both graphs.

**Figure 5:**
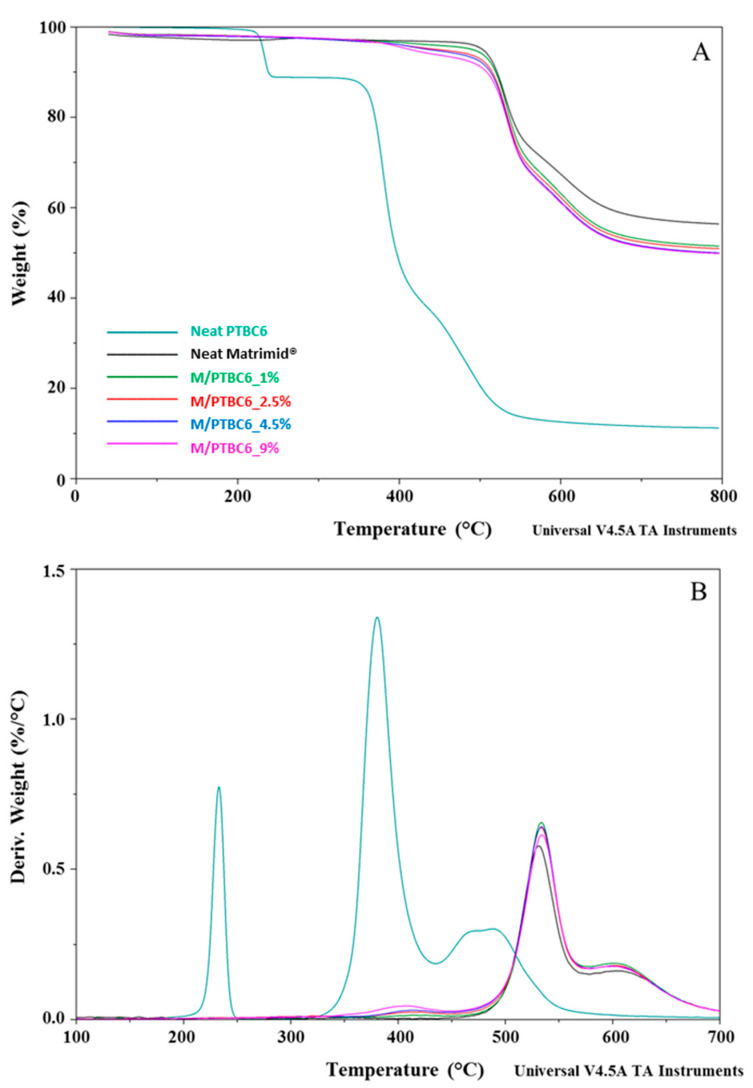
TGA (**A**) and DTG (**B**) curves of the PTBC6, neat Matrimid^®^ and Matrimid^®^/PTBC6 blends. The legend refers to both graphs.

**Figure 6:**
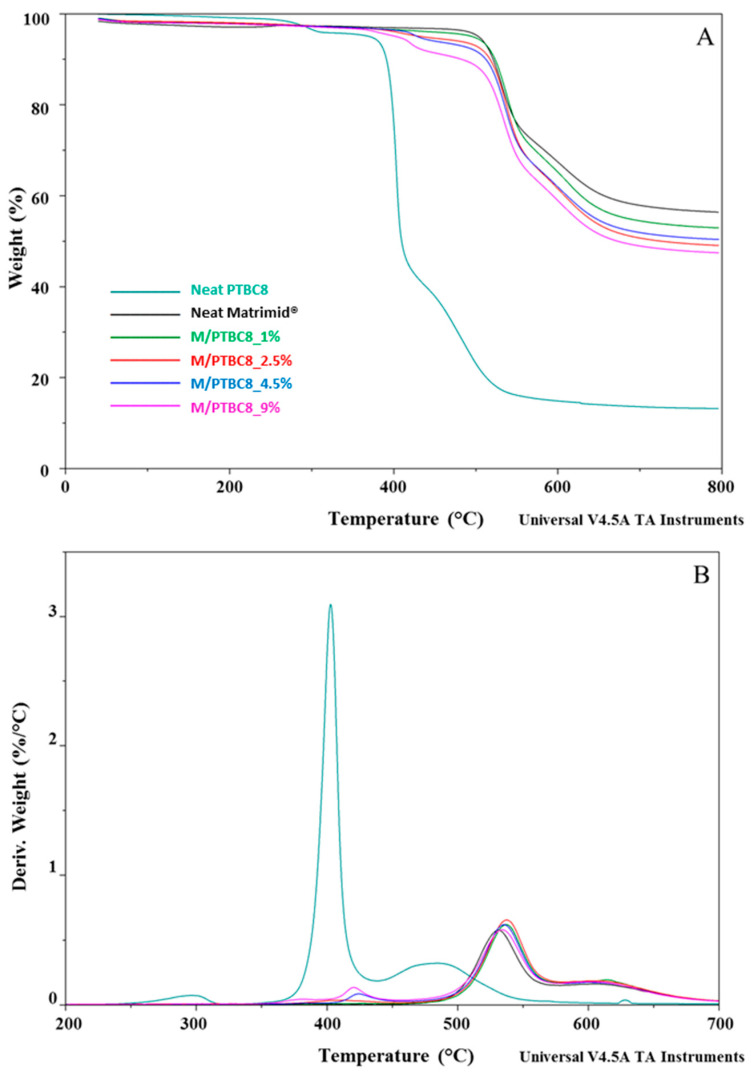
TGA (**A**) and DTG (**B**) curves of the PTBC8, neat Matrimid^®^ and Matrimid^®^/PTBC8 blends. The legend refers to both graphs.

The thermostability of neat additives, neat Matrimid^®^ and M/PTBC blends was assessed by TGA analysis at a heating rate of 10 °C/min under a nitrogen atmosphere. TGA and DTG curves of PTBC4, PTBC6 and PTBC8 are reported in Figure 3, while those obtained on pure Matrimid^®^ film are compared to the nanocomposite films in Figure 4, Figure 5 and Figure 6. 

The temperature corresponding to the 5% weight loss was considered as the onset decomposition temperature (*T*onset) to avoid the uncertainty from the manual determination of *T*onset as intersection of the starting mass baseline and the tangent to the TGA curve at the point of maximum gradient, as previously reported [24]. Considering the temperature describing the decay onset (*T*onset of 173 °C, 231 °C and 370 °C, for PTBC4, PTBC6 and PTBC8, respectively), the thermostability of the calixarenes was found to improve as the number of phenyl groups increases (Figure 3A). It could be related to the hydrogen bonds between the hydroxyl groups in the calixarenes. 

Three degradation steps are evident in all the calix[n]arenes studied (Figure 3A). Pyrolysis studies evidenced that the decomposition of the PTBCs involves the loss of *tert*-butyl groups caused by the instability of the methyl group, while the second and third stage of pyrolysis are the autopyrolysis of its bridged methylene and benzene rings, respectively [25].

The three degradation steps of PTBC4 were found in the temperature ranges of 130–218 °C, 236–415 °C and 425–548 °C, respectively, and the decomposition was almost complete at ca. 550 °C (weight loss 96%), as previously reported [26]. The second and third decomposition stages of the other calix[n]arenes are located at higher temperatures according to their larger size (degradation temperature in the ranges of 310–435 °C and 346–435 °C and of 437–586 °C and 438–568 °C for PTBC6 and PTBC8, respectively). Unlike PTBC4, at the end of the three degradation steps, a reduced mass loss was observed for PTBC6 (76%) and PTBC8 (84%), both showing residues above 10% at 800 °C. The residue obtained at 800 °C under N_2_ atmosphere corresponds to the char yield. A reduced weight residue could be found performing the analysis in air [27].

The DTG curve of PTBC4 (Figure 3B) showed a peak at 173 °C (*T*d1), a second peak at 346 °C (*T*d2) and a less noticeable peak at 479 °C (*T*d3). Similarly to PTBC4, PTBC6 and PTBC8 display a *T*d1 peak (at 233 °C and 297 °C, respectively) that could be due to residual solvent or guest molecules traces. The same trend of increased *T*onset was observed for the *T*d2 and *T*d3 comparing the different calixarenes. *T*d2 was found at 380 °C in PTBC6 and at 403 °C in PTBC8 (310–435 °C and 346–435 °C ranges, respectively), showing 41% and 55% degradation percentages, respectively. *T*d3 was shifted to 495 °C in PTBC6 and to 492 °C in PTBC8 (temperature ranges of 437–586 °C and 438–568 °C, respectively). The decomposition percentage during the third step was ca. 25% for both PTBC6 and PTBC8, confirming their higher thermal stability with respect to PTBC4.

The thermal decomposition had two steps in the Matrimid^®^ film, whereas it became multistep in the nanocomposite films (Figure 4, Figure 5 and Figure 6). The high thermal stability of Matrimid^®^ is indicated by a *T*onset at 503 °C, a main degradation peak (*T*d1) at 530 °C and a second decomposition one (*T*d2) at around 609 °C, which is related to the thermal decomposition of the imide group and carbonization [28]. Nevertheless, at 800 °C, a residue of 56% was registered, indicating that higher temperatures are required for a complete degradation of the polyimide.

A decrease in *T*onset in the blends, according to calixarenes ring size and concentration onto polymer matrix, was observed. However, only minor initial mass losses were present in the blends. In particular, a high decrease in *T*onset was observed in the M/PTBC4 membranes, showing values ranging from 352 to 341 °C as filler concentration increases from 2.5% to 9%. These variations were less marked in M/PTBC6 and M/PTBC8 blends (decreasing values from 491 to 413 °C and from 495 to 402 °C as additive concentration increases from 1% to 9%). 

The *T*onset decrease registered in all blends, particularly with the highest (9%) additive concentration, was not observed in *T*d1 and *T*d2. Specifically, the *T*d1 peak showed an increase of ca. 4 °C in M/PTBC4, of max 3 °C in the M/PTBC6 and in the range 3–6 °C in M/PTBC8 blends, respectively, as also observed using MOFs as a filler [29]. Considering the *T*d2, the maximum decrease (6 °C) was detected in the M/PTBC6 9% blend, while a decrease of max 3 °C was observed in M/PTBC4, whereas no significant variations were observed in the M/PTBC8 blends except for the increase of 6 °C in the M/PTBC8 1% membrane, confirming the better contribution to polymer stability of this filler. Furthermore, all blends showed another degradation peak before *T*d1 in the ranges of 291–424 °C (M/PTBC4), 400–408 °C (M/PTBC6), and 382–424 °C (M/PTBC8). This peak depends on the degradation of the PTBCs that occurs at temperatures considerably lower (by more than 100 °C) compared to Matrimid^®^, indicating that the filler decomposition does not affect the main degradation step (*T*d1) of the blends (Figure 4B, Figure 5B and Figure 6B). These peaks are not visible at 1% concentration of calix[n]arenes due to the low content of the additives. Considering the residue recorded at 800 °C, a reduction up to 6% and 8%, as the additive concentration increases, was observed in M/PTBC4–M/PTBC6 and M/PTBC8 membranes, respectively.

### 3.2. DSC

Differential Scanning Calorimetric measurements were performed on neat additives, neat Matrimid^®^ and M/PTBC blends to assess the glass-transition (*T*g) and melting (*T*m) temperatures, during the heating runs, and the crystallization (*T*c) temperature, on the cooling scan. Data of cooling and second heating runs, carried out under nitrogen atmosphere at 10 °C/min from −90 to 350 °C, were analyzed.

DSC curves, through the melting point, indicated that the thermal stability of the crystals of PTBC4, PTBC6 and PTBC8 is over 340 °C. The analysis of neat PTBC4 evidenced, on cooling and second heating runs, exothermic (*T*c) and endothermic (*T*m) peaks at 325 °C and 341 °C, respectively (Figure S3). The melting point is in accordance with the manufacturer’s data (*T*m ≥ 300 °C) but lower than that detected by Deligöz et al. [30] for free Calix[4]arene (351 °C, peak point). 

None of these peaks were found in the DSC curves of PTBC6 and PTBC8 in the investigated temperature range (Figure S4). Indeed, both additives have *T*m above 350 °C, as reported by the producer and in the literature [26,31].

**Figure 7:**
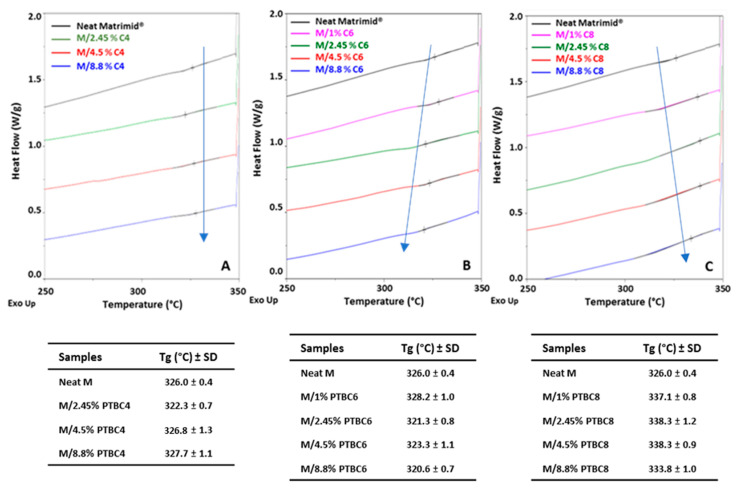
Overlay of DSC curves obtained on Matrimid^®^ and Matrimid^®^/PTBC composite membranes in the second heating scan. (**A**) PTBC4; (**B**) PTBC6; (**C**) PTBC8.

The DSC traces obtained for the PTBC-loaded membranes are gathered in Figure 7 and compared to the neat polyimide. The analysis of neat Matrimid evidenced the *T*g at 326 °C, which is in agreement with Venna et al. [29]. This value is higher than those reported in other papers (310–319 °C) [20,32,33] due to variables involved in the film production methods (solvent, temperature, drying steps, etc.). 

The loading of the calix[n]arenes into the selected polyimide determined a different behavior according to their ring size. 

M/PTBC4 blends (Figure 7A) showed similar *T*g values to that of neat Matrimid^®^ except for the 2.5% concentration, which had a slight decrease in *T*g. Thus, the membrane rigidity remained almost constant, indicating a poor compatibility between the two components.

M/PTBC6 blends (Figure 7B) presented a *T*g decrease (2–5 °C) in the blends loaded with concentrations in the range 2.5–9%, indicating that the polymer matrix became slightly more flexible, whereas at low concentration (1%), a slight increase was registered.

Instead, *T*g values higher than that of neat Matrimid^®^ were observed in the M/PTBC8 blends (Figure 7C), showing that polymer stiffness increases as a result of the interaction between polymer chains and PTBC8. Other studies reported a similar increase in the *T*g upon the incorporation of solid particles in Matrimid^®^, with consequent rigidification at the polymer/filler interface due to the interaction between particles and the polymer, limiting the mobility of the polymer chains adjacent to the fillers [29,34]. Conversely, blending a soft polymer such as PEG with Matrimid^®^ improved the local chain flexibility as evidenced by a reduced *T*g value [32]. Moreover, the increase in the calix[n]arene concentration and the occurrence of a larger end-to-end chain distance could be related with the increased polymer chain’s rigidity, as predicted in a simulation study on M/PTBC4 membranes [20].

### 3.3. FT-IR

**Figure 8:**
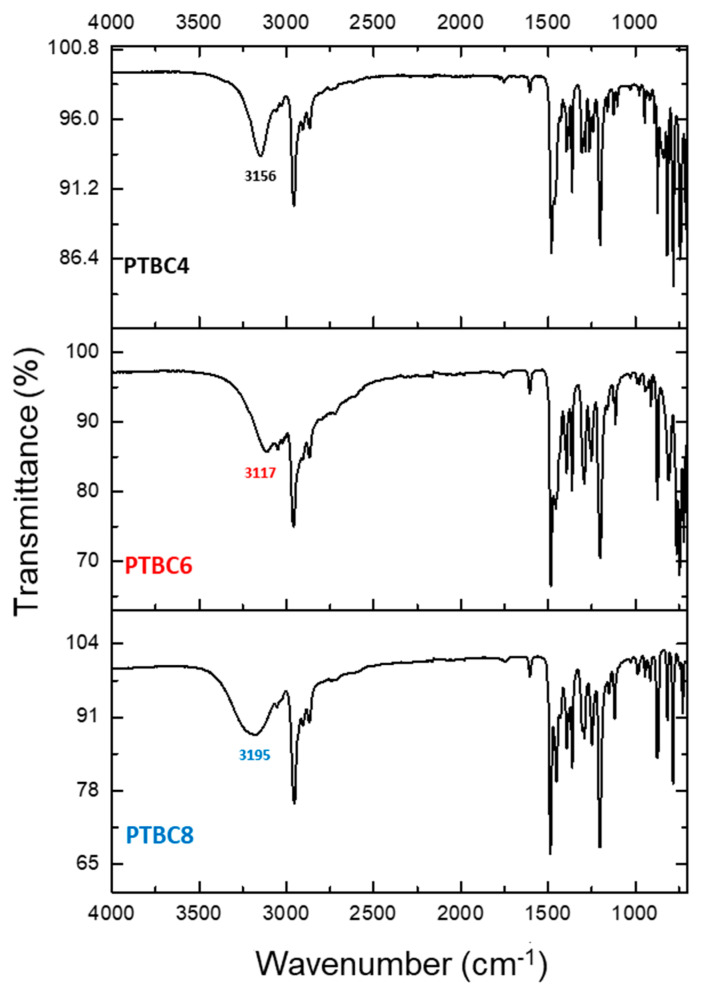
FTIR-ATR spectra of PTBC4, PTBC6 and PTBC8.

The ATR–FTIR spectra for the PTBC macrocycles did not show the band of free OH groups that is typically found at ca. 3600 cm^–1^ (Figure 8). Instead, the distinctive low-frequency stretching vibration of the phenolic OH was evident in the range from 3120 to 3200 cm^−1^, depending on the size of the macrocycle. The shift is strongest in PTBC6, medium in PTBC4 and weakest in PTBC8, according to the literature data [35]. It depends on very strong intramolecular hydrogen bonds between the hydroxyl groups in PTBC [36]. Furer et al. evidenced this behavior for PTBC8, concluding that all eight hydroxyl groups are involved in H-bonds, leading to the “pleated-loop” conformation [37]. The band at ca. 1470 cm^–1^ corresponds to the asymmetric deformation vibrations of the methylene groups, while that at ca. 1600 cm^–1^ results from the bending vibrations of aromatic CCH [38]. 

**Figure 9:**
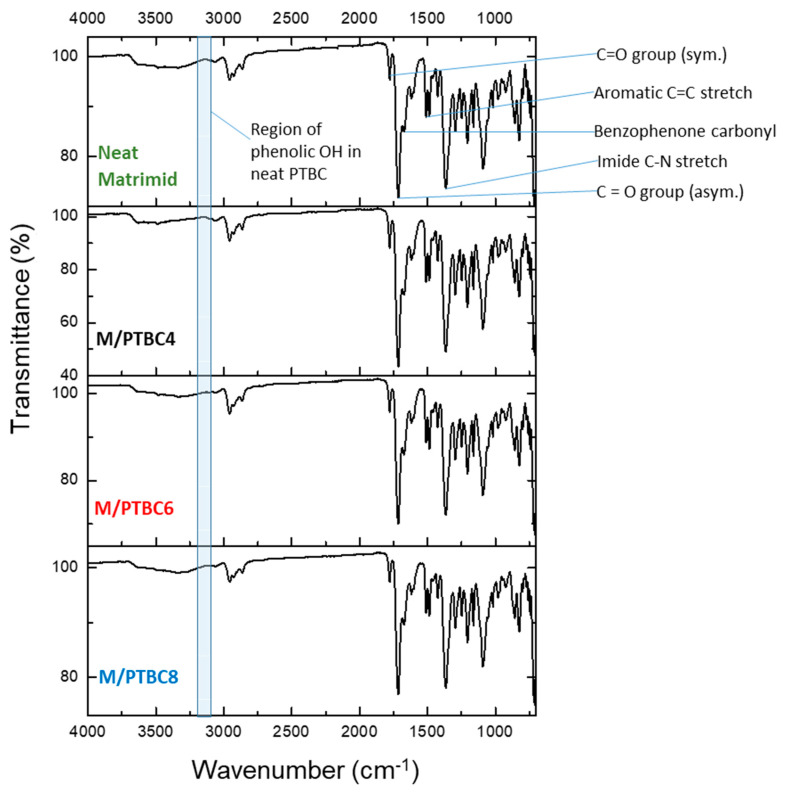
FTIR-ATR spectra of pristine Matrimid^®^ membrane and of samples loaded with 9 wt% of PTBC4, PTBC6 and PTBC8.

The spectra of pristine Matrimid^®^ and of the films loaded with 9 wt% of PTBC4, PTBC6, or PTBC8 are shown in Figure 9. All characteristic imide bands were maintained in the M/PTBC films. The stretch vibration for the C=O group in five-membered cyclic imide rings (anti-symmetric stretch vibration of carbonyl in the ketonic group) is at 1778 cm^–1^ in the spectrum of pure Matrimid^®^. Similar values were observed for the nanocomposite films. The corresponding symmetric stretch was observed at 1713 cm^–1^ for pure Matrimid^®^ and at 1715–1716 cm^–1^ for the PTBC-loaded Matrimid^®^. 

The symmetric stretch vibration of benzophenone carbonyl in the imide of pristine Matrimid^®^ observed at 1671 cm^–1^ was shifted toward higher wavenumber range in the PTBC-loaded polymeric matrix (1672–1674 cm^−1^). The C-N stretch of the imide group of Matrimid^®^ was detected as a sharp absorption band at 1365 cm^–1^ and at 1367–1366 cm^–1^ for the PTBC-loaded polymeric matrix. The stretching frequencies of aromatic double bonds (C=C) were observed at 1512 cm^–1^ with a slight shift toward lower wavenumbers (1509–1511 cm^–1^) in the PTBC-loaded Matrimid^®^. The peaks at 1487 and 1617 cm^−1^ are the stretching vibrations of aromatic double bonds. The presence of the aromatic C=C groups of the calixarenes is shown by a more intense absorbance at 1487 cm^−1^. The N–H peak at 3490 cm^−1^ in Matrimid^®^ has a decreased intensity in the nanocomposite membranes due to overlapping of the PTBCs O–H stretching band. 

The detected small shifts (see Table 2) indicate slight chemical interactions between polymer/additive.

### 3.4. NMR Analysis

The proton NMR spectra of the film M/PTBC8–9 wt%, dissolved in deuterated chloroform as a solvent, showed the typical signals of the two components, without significant chemical shift changes compared to the spectra of Matrimid^®^ and PTBC8 alone in the same solvent (Figure S2). This result was consistent with the slight chemical polymer/additive interactions observed in the FT-IR spectra.

### 3.5. Membrane Morphology

Macroscopically, the nanocomposite specimens kept the original transparency, partial flexibility and the yellowish coloration that is characteristic of the neat polymer matrix (Figure S5). The used additives did not induce observable defects in the films. However, as assessed by gas permeation tests, the PTBC4 loading resulted in defective samples, while PTBC6 and PTBC8 produced defect-free samples. The membrane thickness was in the range of 30–35 microns, as also confirmed by SEM analysis.

**Figure 10:**
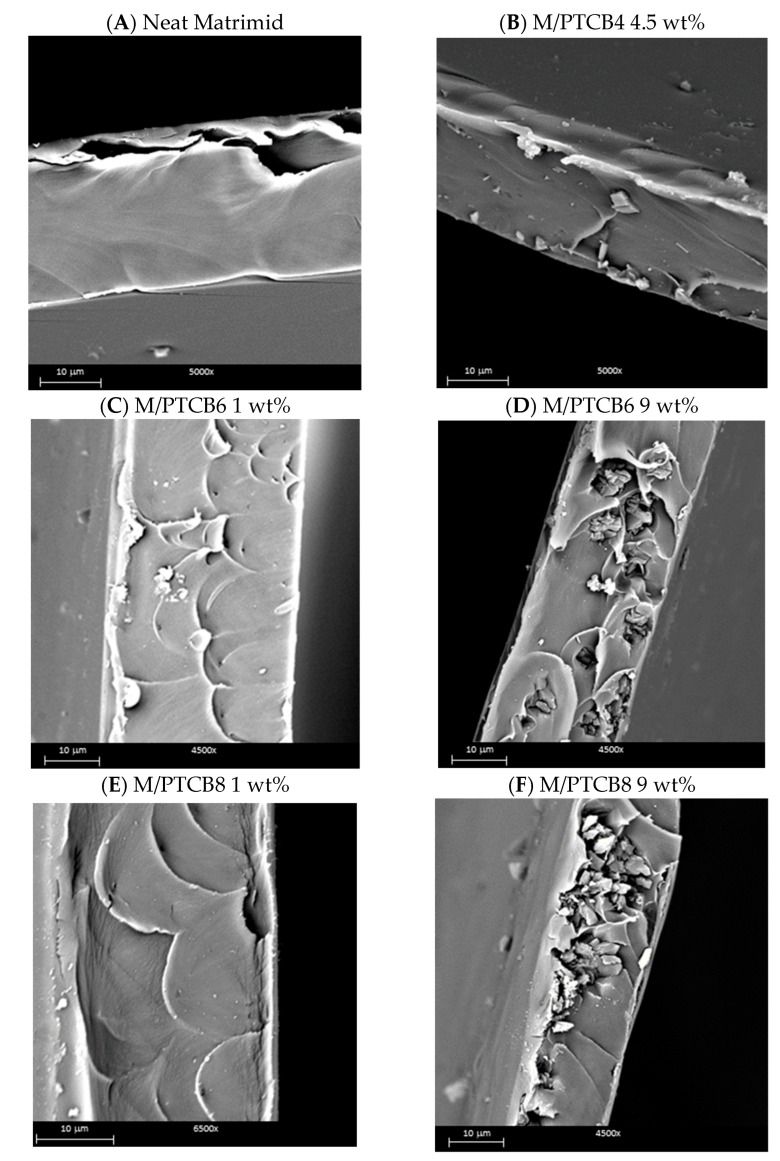
SEM micrographs of the prepared membranes, cross-section. (**A**) Neat Matrimid; (**B**) M/PTCB4_4.5 wt%; (**C**) M/PTCB6_1 wt%; (**D**) M/PTCB6_9 wt%; (**E**) M/PTCB8_1%; (**F**) M/PTCB8_9%.

Figure 10 gathers representative micrographs obtained on the cross-section of the prepared samples at different PTBC concentration. The visible micrometer-sized cavities could be generated by the ductile fracture mode of the membranes due to fracturing stress in liquid N_2_, as already described in the literature [39]. 

In contrast with the perfectly smooth neat Matrimid membrane surface, samples incorporating fillers show a rougher surface with the presence of isolated lumps facing the surface (see Figure S6). Concerning the filler distribution along the section, it is evident that the PTBC4 particles are only partially wrapped in the polymer matrix. 

The scalloped morphology observed for the MMMs doped with PTBC6 and PTBC8 (Figure 10C,E) was attributed to the formation of elongated polymer segments having increased plastic deformation [29]. The crater-like morphology is evident in the cross-section of M/PTBC at low loadings, which is not more present as filler concentration increases. This texture is typical for Matrimid-based MMMs when a good compatibility between filler and polymer phases is achieved [40]. 

As the additive concentration increases, the formation of aggregates, in which particles maintain their distinct characteristic appearance, is visible. These assemblages are distributed non-uniformly in the various areas of the membrane, particularly in the case of PTCB8. Therefore, as observed in Figure 10F for PTCB8 loading of 9%, the aggregation of the particles near the surface of the membrane results in a “*sieve-in-a-cage* morphology” [41]. The resulting formation of by-pass at the particle–polymer interface in the presence of voids larger than permeant molecules becomes inevitable, producing defects in the membranes. This is further supported by the gas permeation tests and discussed in the next section. 

In contrast, the PTCB6 samples, although suffering from the same phenomenon at high concentration, showed less concentrated aggregates surrounded by polymer with greater chances of membrane integrity for gas transport (see Figure 10D). 

### 3.6. Gas Permeation

#### 3.6.1. Effect of the Additive Type and Loading

At a fixed temperature, the gas permeability depends on the nature of the polymer matrix (e.g., rigidity and eventual crystallinity of the polymer), on the filler type and concentration, on the polymer/filler interactions, and on the permeant molecules. In particular, owing to their small molecular size (<1 nm), the permeation of the selected permanent gases can elucidate the membrane microstructure.

The three additives differ for their rigidity. It is known that among PTBC4, PTBC6 and PTBC8, the PTBC4 macrocycle is the most rigid oligomer [35]. PTBC4 can assume four different conformations, but preferentially, it adopts a cone shape conformation, which is stabilized by a cyclic array of OH group intramolecular hydrogen bonds [42]. The conformational blockage is also due to the hindered rotation of the aromatic rings through the small calixarene cavity, the so-called ‘*through the annulus tert-butyl passage*’. Differently, the larger PTBC6 and PTBC8 oligomers are characterized by a higher conformational mobility due to the rotation of both OH and *tert*-butyl groups through the larger cavities [17]. This can be the reason for the poor compatibility of PTBC4 with the host matrix. Thus, a ‘*sieve-in-a-cage*’ structure, with leaky interfaces, can be inferred for the Matrimid/PTBC4 membranes as also evidenced by SEM images (Figure S6). Such undesirable morphologies, due to the lack of interfacial compatibility between the membrane phases, are common issues occurring when inorganic fillers are loaded within an organic polymer, as reported for the 5A zeolite enclosed in Matrimid^®^ [43]. 

The glassy nature of the used polyimide makes the incorporation of a rigid filler difficult, even if organic, compared to a flexible and rubbery matrix as Pebax [18].

The single gas permeation parameters of the ‘as-prepared’ samples, reported in Table 3 as individual gas permeability and in Table 4 as selectivity for certain gas pairs, are compared to the pristine polyimide films.

The gas permeation order that characterizes the neat polyimide (H_2_ > He > CO_2_ > O_2_ > N_2_) was maintained in the nanocomposite samples. Thus, the process is ‘diffusion controlled’ in the neat Matrimid^®^ and in the nanocomposite films. Accordingly, the MMMs displayed noteworthy selectivity values for gas pairs that differ in the molecular size (e.g., H_2_/N_2_, He/N_2_) but also in solubility (CO_2_/N_2_). 

**Figure 11:**
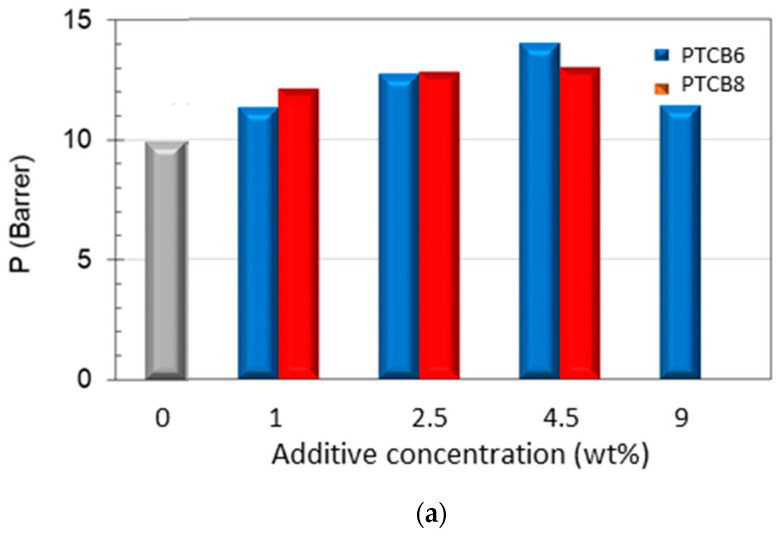

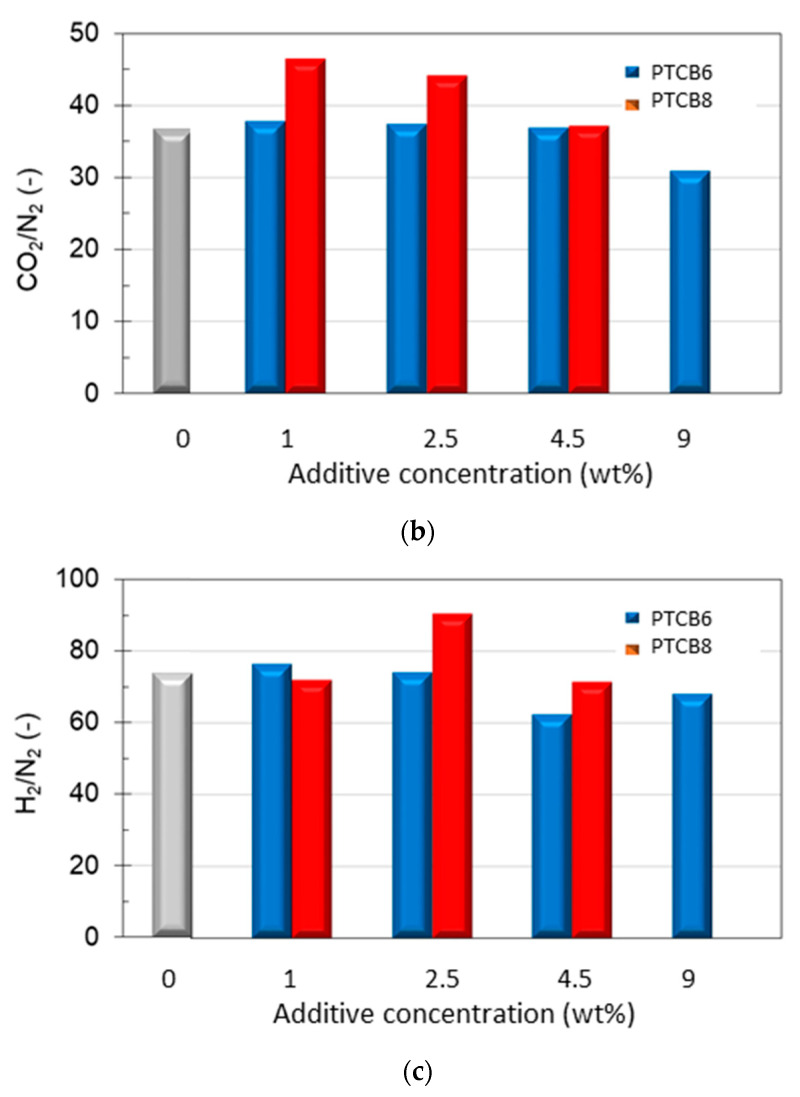
Permeation properties measured in samples of Matrimid^®^ and PTBC8 or PTBC6 at various loadings. (**a**) Permeability of CO_2_, (**b**) CO_2_/N_2_ selectivity, (**c**) H_2_/N_2_ selectivity.

The analysis of the PTBC loading effect on the gas permeation revealed an enhanced permeability at increasing PTBC6 or PTBC8 concentration compared to the neat polyimide. The largest increment in permeability was obtained in the nanocomposites loaded with PTBC6 (Figure 11a). However, the permeability gain depends on gas type and on filler loading. The trends were not monotonous with a maximum at a concentration of 4.5 wt% for carbon dioxide (Figure 11a) and at 2.5 wt% for small molecules (e.g., helium and H_2_). 

The increase in permeability was more important for CO_2_, which is the more interactive species with the polymer matrix. Once the permeability of CO_2_ in the pure polymer (*P*_0_) is set equal to one, the percentage increase in the gas permeability with the amount of filler incorporated into the matrix varied, as reported in Figure S7. Increases in permeability over 140%, combined with negligible changes in CO_2_/N_2_ selectivity, can be achieved in samples containing 4.5 wt% of PTBC6, whereas less pronounced permeability increases (+120%) linked to a significant rise in CO_2_/N_2_ selectivity in samples containing down to 2.5 wt% of PTBC8 were observed. 

On the other hand, CO_2_ molecules, having a quadrupole moment, make strong interactions with the oxygen atoms of the calixarene hydroxyl groups. Consequently, the CO_2_ selectivity was improved, particularly using PTBC8 at low concentration (Figure 11b). 

Interestingly, the nanocomposites containing PTBC8 presented a gain in both permeability and selectivity values for CO_2_/N_2_ pair with respect to the neat polymer. This behavior is opposed to the common trade-off between permeability and selectivity observed in polymeric materials. However, the excellent CO_2_/N_2_ selectivity tends to decrease as the PTBC8 content increases, reaching the intrinsic selectivity of the neat polymer (Figure 11b). At the highest examined additive concentration (9 wt%), the PTBC8 membranes became defective, showing the typical Knudsen selectivity values (not shown in Figure 11). Instead, membranes containing the same amount of PTBC6 remained moderately selective but less permeable than those prepared at lower concentrations (Table 4).

Thus, the permeation tests evidenced that the PTBC6 and PTBC8 macrocycles created additional permeation paths for the gas transport. This positive effect on the permeability can be related to the porous nature of the fillers and/or to a spacing effect exerted on the polymer chains by the larger and flexible macrocycles. 

The inner cavity diameter and molar volume increase from PTBC4 to PTBC6 to PTBC8 [44]. The cavity size can vary depending on the conformation adopted by the macrocycle. When the solvent cannot form a hydrogen bond with the OH groups of the calixarene and all the OH groups are intramolecularly hydrogen bonded in a cyclic array, a cone, a *pinched-cone* and a *pleated-loop* (Figure 2) are the main conformations adopted by PTBC4, PTBC6 and PTBC8, respectively, with an inner cavity diameter ranging from around 3 Å for the smallest to 7−8 Å for the largest macrocycle [45]. 

Despite the larger cavity size, PTBC8 produces the highest permselectivity values. DSC demonstrated pronounced polymer/filler interactions in the M/PTBC8 samples leading to an increased rigidity for the polymer matrix. In addition, the large PTBC8 cavity could be partially occluded by the polymeric chains, thus producing more selective pathways in the membrane, as also evidenced by comparing the permeability of small and large molecules (e.g., H_2_/N_2_). TGA did not evidence the presence of low boiling point solvents as were used in the membrane preparation (DCM). Thus, a reduced cavity occupancy that can occur due to the polarity of the DCM, as reported in a study for PTBC6 [15], can be excluded. 

Although demonstrating less effectiveness in improving selectivity, PTBC6 macrocycles were more compatible with the selected polyimide, enabling a greater loading of the filler to be incorporated within the polymeric matrix. This result was consistent with the higher conformational flexibility of PTBC6. NMR studies [10] have shown that PTBC4 and PTBC8 have very similar resonance spectra in non-polar solvents, which is consistent with structures blocked by intramolecular hydrogen bonding, whereas PTBC6 seems to be more flexible due to the mobility of the methylene bridges which can be positioned inside and/or outside the plan formed by the phenolic groups [37]. The flexibility could result in a better adaptability of the PTBC6 with the polymeric matrix as also confirmed by DSC results. 

The enhanced permeability in the nanocomposite samples is due to the increase in the apparent gas diffusion coefficients for all gases tested (Table 5). Gas diffusion in M/PTBC6 samples increased with the loading, while M/PTBC8 membranes showed a reverse behavior. This evidence could be an indication of the stacking of the PTBC8 structures in the membranes, which also affects the gas transport. Indeed, these macrocycles are capable of forming chain-packing motifs [46].

The observed enhanced permeability of the membranes enclosing the PTBC calixarenes differs from the behavior reported for membranes based on the highly permeable PTCNSi1 polymer loaded with similar fillers [17]. A reduction in the free volume upon the loading of the calixarenes was assessed by probing the microstructure of the PTCNSi1 membranes by PALS [17]. In a highly permeable matrix such as the PTCNSi1, the calixarene molecules could fill the free volume elements, thus depressing the gas permeation fluxes. Instead, in a polymer with smaller free volume elements such as the selected polyimide (Matrimid^®^), this occurrence can be avoided. 

A macroscopic modeling study was carried out to correlate the gas permeability data to the membrane structure. The specific molecular interactions evidenced by FT-IR were not so strong. Consequently, a ‘separate phase’ approach was considered adopting the Maxwell model [47]. It describes the nanocomposite membrane as a matrix of one component (polymeric phase) with the other one (PTBC fillers) as a dispersed phase (Equation (4)):

(4) $$P_{n}=P_{c}\;\cdot \;\frac{P_{d}+2P_{c}-2\phi_{d}\;\left(P_{c}-P_{d}\;\right)}{P_{d}\;+2P_{c}+{\;\phi }_{d}\;\left(P_{c}-P_{d}\;\right)}$$

where the subscripts *n*, *c* and *d* represent the nanocomposite film, the continuous matrix and the dispersed phase, respectively, while ϕ is the volumetric filler loading.

The volumetric fraction of the fillers was obtained considering a density of 1.24 g/cm^3^ for the polymer and 1.1 g/cm^3^ for the fillers. 

**Figure 12:**
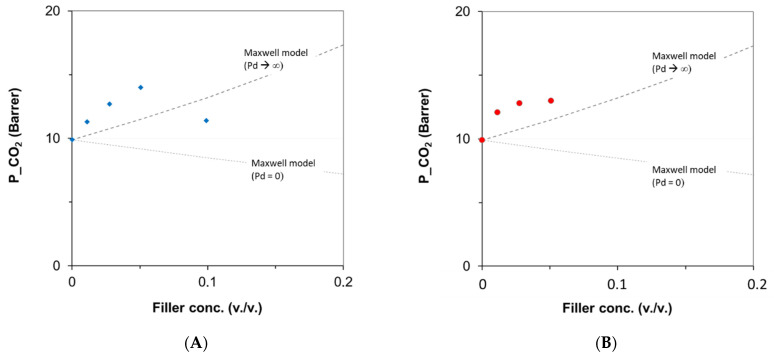
CO_2_ permeability of Matrimid MMMs versus the content of PTBC fillers. Lines represent the Maxwell models; symbols are the experimental data. (**A**) M/PTBC6; (**B**) M/PTBC8.

The CO_2_ permeability, reported as a function of the PTBC volumetric concentration in the membranes, was compared to the theoretical predictions (Figure 12). The experimental points were above the limit predicted by the Maxwell model for nanocomposites enclosing a filler having a permeability much higher than that of the polymeric matrix (*P*_d_ >> *P*_c_). Since these data exceed the upper limit identified by the model, the great potential of the PTBC incorporated in the Matrimid matrix was confirmed. A better representation could be obtained by removing the assumption of spherical fillers and considering a different shape factor. However, for each filler type, a single model equation cannot represent the data obtained at increasing contents of the PTBC fillers. At larger concentrations, non-ideal effects affect the gas transport (e.g., agglomeration), resulting in the downward trend for the experimental points.

#### 3.6.2. Effect of the Temperature on the Gas Permeation

A progressive increase in permeability and diffusivity with temperature occurred for all gases in the range 25–55 °C. However, the gas permeation order remains the same as that of neat polymer. 

The gas permeation parameters enhanced according to the Arrhenius law:

*P* = *P*_0_ exp(−*E*_P_/RT) (5)

*D* = *D*_0_ exp(−*E*_D_/RT) (6)

where *P*_0_ and *D*_0_ are pre-exponential factors, *E*_P_ and *E*_D_ are the apparent activation energy for permeation, *R* is the ideal gas constant and *T* is the temperature. 

The activation energy was calculated for the different samples as the slope of the permeability logarithm versus the reciprocal of absolute temperature (Table 6).

In the neat Matrimid^®^, the activation energy of permeation for all gases, except for CO_2_, increased with increasing gas molecular size. This indicates a strong size-sieving behavior [48]. This trend was maintained for all samples. Considering that permeability can be decoupled in kinetic (*D*) and thermodynamic (*S*) terms, the specific contribution of each parameter affects the final response to the temperature change. The diffusion of small molecules was three orders of magnitude higher than that of larger species; therefore, they also show higher permeability values despite having the lowest solubility. 

On the other hand, CO_2_ showed the lowest activation energy of permeation for each of the prepared samples. Indeed, among the gases considered, CO_2_ is the most condensable and soluble. Its linear shape facilitates its diffusion. It has the most exothermic (i.e., negative) enthalpy of sorption, resulting in a lower *E*_P_. Thus, chemical interactions between CO_2_ and polar groups in the membranes cause more exothermic sorption [49]. 

The activation energy of permeation process (*E_P_*), measured for partly aged samples (3 months), did not change dramatically in the loaded samples with respect to neat polymer (Table 6). Thus, the additives did not oppose restriction to the gas molecules.

**Table 6:** Activation energy for permeation on the neat Matrimid^®^ and on the MMMs with 4.5% of PTBC fillers (aged samples, 3 months).

Membrane Code	Activation Energy for Permeability, *E*_P_ (kJ/mol)					Ref.
	H_2_	He	CO_2_	O_2_	N_2_	
Neat Matrimid^®^			13.0			[50]
Neat Matrimid^®^	11.34		9.03		19.7	[51]
Neat Matrimid^®^		13.6	9.8		20.7	[52]
Neat Matrimid^®^	11.7	12.5	8.2	16.6	25.6	This work
M/PTBC6, 4.5 wt%	13.7	12.5	7.9	13.1	21.1	This work
M/PTBC6, 4.5 wt%	12.8	12.9	8.3	15.2	22.4	This work

On the other hand, significant differences were observed in *E*_D_ (Table 7), particularly for small and less interacting gases. In particular, for helium, a halving was measured in filled samples, and for hydrogen, a reduction of about one-third of the value calculated for neat polymer occurred. Thus, a more stable behavior with temperature for the diffusion of small gases through the nanocomposite membranes is obtained. Concerning nitrogen in the presence of the fillers, a reduction of about 10 wt% for *E*_P_ is accompanied by a comparable increase in *E*_D_. Thus, the nitrogen solubility is increased in the nanocomposite membranes.

Therefore, in these nanocomposite membranes, the temperature caused an increase in permeability mainly due to the diffusion coefficient (exothermic process), which is partly moderated by the solution (endothermic process). 

**Figure 13:**
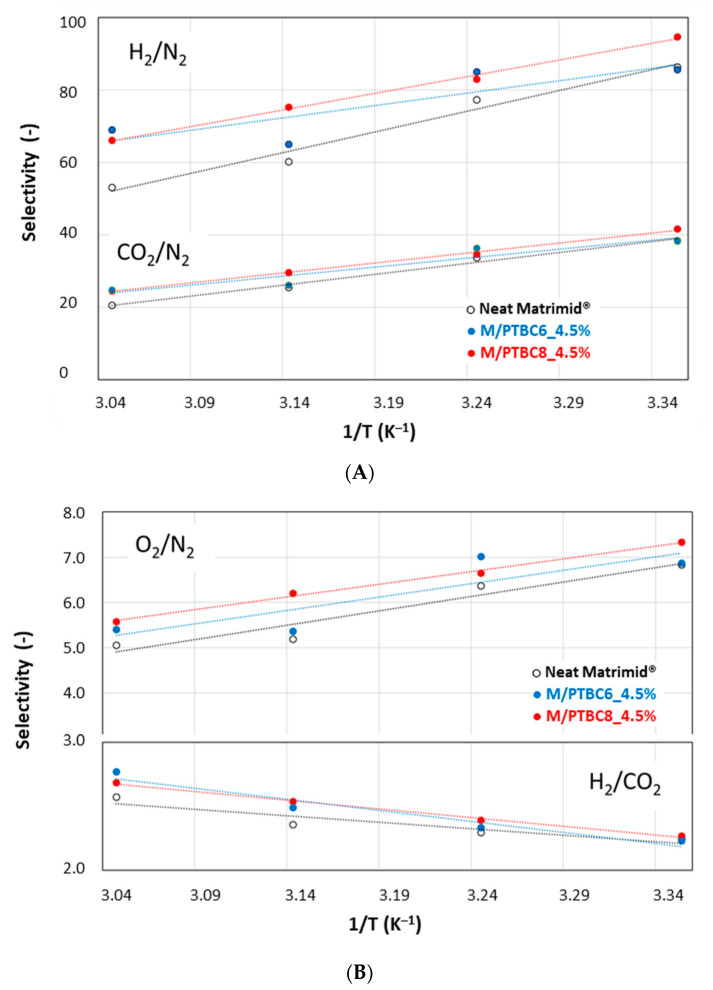
Selectivity of Matrimid MMMs versus the temperature reciprocal. Symbols are the experimental data; lines represent the Arrhenius equation. (**A**) H_2_/N_2_ and CO_2_/N_2_; (**B**) O_2_/N_2_ and H_2_/CO_2_.

A general decrease in gas selectivity values was observed for all gas pairs, as the less permeable species are more favored by an increase in temperature. On the other hand, a moderate increase for the H_2_/CO_2_ selectivity was observed by increasing the temperature (Figure 13). In any case, in the whole investigated range of temperature, an improvement in gas selectivity was observed in the presence of the additives, particularly with PTBC8. Indeed, the data for the M/PTBC8 move parallel and only shifted upwards with respect to the values of the neat sample.

#### 3.6.3. Aging Behavior

**Figure 14:**
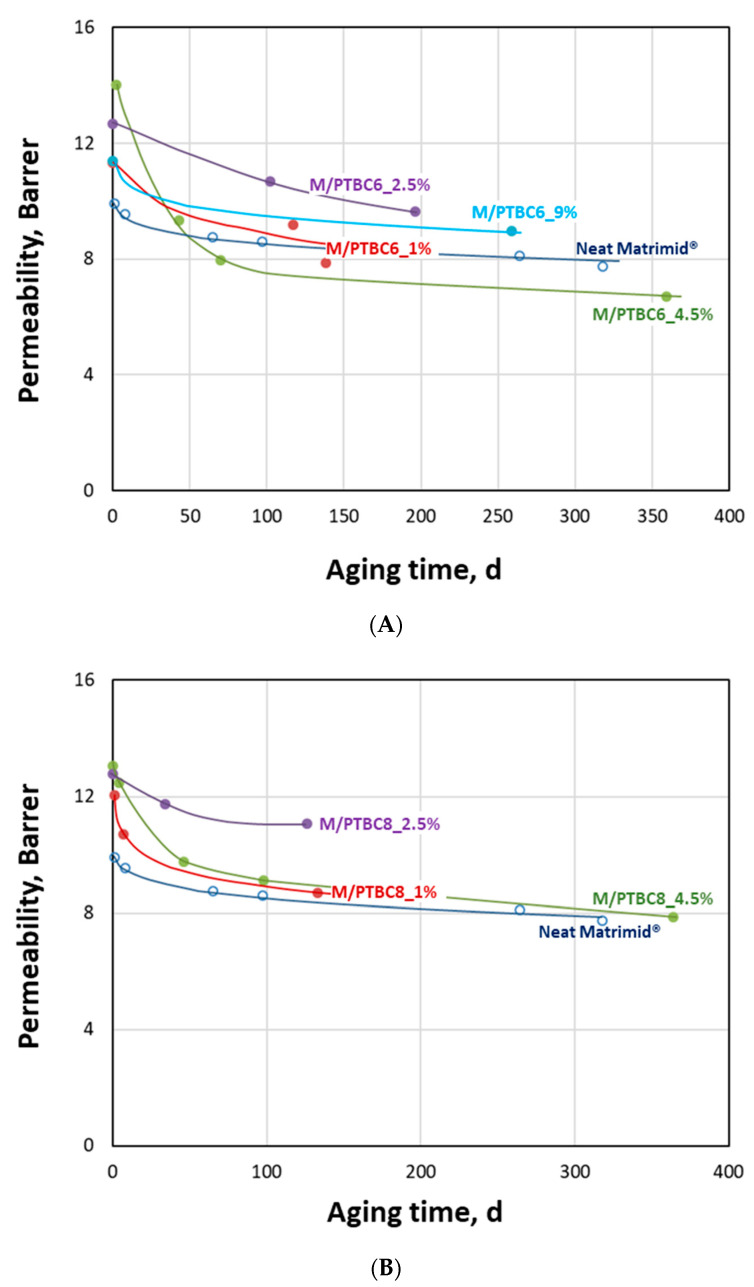
Aging behavior of the Matrimid^®^-based membranes. CO_2_ permeability versus aging time since the membrane preparation. Lines represent a guide to the eye. (**A**) PTBC6-loaded membranes; (**B**) PTBC8-loaded membranes.

Achieving constant performance represents an important requirement for the application of membrane systems in separations of industrial interest. For this reason, some membrane samples were also characterized in order to study how gas transport changes over time. A generalized decrease in the CO_2_ permeation rate, combined with a substantial maintenance of the initial CO_2_/N_2_ selectivity, can be observed in all investigated samples (Figure 14).

This behavior is a typical feature for glassy polymers and is due to the loss of ‘extra’ free volume during the rearrangement of polymer chains in a non-equilibrium state [53,54]. It is particularly important in the first periods after the membrane preparation and then tends to “plateau” values at long lifetimes. However, compared to the neat polyimide, the M/PTBC samples tend to preserve their higher gas permeability in the first 150–200 days, depending on their loading. Membrane samples containing 2.5 wt% of PTBC6 or PTBC8 seem to resist better to the aging for at least six months. Nevertheless, the most permeable membranes (containing 4.5 wt% of additives) at long times (about one year) approached the performance of the neat polymer, losing their advantage because of a more pronounced aging rate. In any case, even with respect to this factor, the PTBC8-loaded membranes perform better than the PTBC6-loaded membranes. Thus, as evidenced by the DSC analysis, constrained surfaces are present in the M/PTBC8 nanocomposites that could reduce densification, resulting in a more consistent permeability [55]. 

Interestingly, the proposed comparison with the literature data of several Matrimid mixed matrix membranes (Table 8) shows that low amounts of PTBC fillers are capable of enhancing the CO_2_/N_2_ separation performance, especially with reference to the PTBC8 samples’ selectivity.

## 4. Conclusions

*p*-*tert*-Butylcalix[n]arenes having different ring sizes (n = 4, 6, 8) were incorporated, in the 1–9 wt% concentration range, into a thermoplastic polyimide (Matrimid^®^ 5218). Dense films were prepared by a pre-mixing approach, according to a solution-casting technique with controlled solvent evaporation. 

Thermal analysis indicates that the good thermal stability of the polyimide matrix is preserved in the nanocomposite films, and the main degradation that takes place at 530 °C in Matrimid^®^ is delayed in the composite membranes, suggesting a reinforcing effect of the additives. 

FTIR spectra show subtle shifts in the characteristic peaks indicating weak interactions in the nanocomposite membranes.

Gas permeation tests of different pure gas species reveal that the gas separation performance depends on the flexibility and on the cavity size of the loaded calixarene. PTBC4 leads to defective samples in all the tested concentrations due to the rigidity of these macrocycles. Instead, the more flexible and larger additives (PTBC6 and PTBC8) have defect-free films that are more permeable than the neat polyimide membranes. 

A maximum in CO_2_ permeability is achieved at a concentration of 4.5 wt% in both cases, while at higher concentration, there is a decay in the membrane-separating performance, which is particularly evident for the larger PTBC8 macrocycle. These results are consistent with the DSC analysis that revealed a reduction in glass transition temperature for PTBC6 and an increase in the films loaded with PTBC8. Accordingly, the medium-size PTBC6, having a better conformational flexibility, can be loaded up to greater amounts without introducing defects in the film. 

The macrocycles 3D open cavities also favor a selective transport of gas molecules, particularly in the case of PTBC8, which results in a simultaneous enhancement for the gas permeability and permselectivity. 

The nanocomposite samples aged for a period of ca. six months keep the higher permeability compared to the neat polyimide.

The gas permeation rates measured as a function of the operation temperature obey Arrhenius’ law, and the diffusion term dominates the gas transport in all the prepared samples. A further enhancement in CO_2_/H_2_ selectivity is observed as temperature increases in the nanocomposite membranes.

## Figures and Tables

**Table 1:** Codes, chemical composition and properties of the studied calixarenes.

Code	Additive Type	Upper Rim	Lower Rim	Density g/cm^3^	Molar Volume cm^3^
PTBC4	Calix[4]arene	t-Bu	(OH)_4_	1.1 ± 0.1	592.6
PTBC6	calix[6]arene	t-Bu	(OH)_6_	1.1 ± 0.1	888.9
PTBC8	calix[8]arene	t-Bu	(OH)_8_	1.1 ± 0.1	1185.1

**Table 2:** Summary of IR spectra values on Matrimid^®^ membrane and on samples loaded with 9 wt% of PTBC4, PTBC6 and PTBC8.

Membrane	Matrimid^®^	M/PTBC4	M/PTBC6	M/PTBC8
Group	Frequency (cm^−1^)			
Polyimide C=O group	1778	1778	1778	1778
Corresponding asymmetric stretch	1713	1715		1756
Imide C-N stretch	1365	1367		1366
Stretching of aromatic double bond	1512	1509		1511
Imidic group	1671	1672		1674

**Table 3:** Permeability of the Matrimid-based membranes, neat and MMMs (‘as prepared’, *T* = 25 °C).

Sample	Conc. (wt%)	Permeability (Barrer)					
		He	H_2_	N_2_	O_2_	CO_2_	CH_4_
Neat Matrimid^®^	0	18.7	19.9	0.27	1.80	9.90	0.25
M/PTBC6	1	20.0	22.9	0.30	1.94	11.3	0.28
	2.5	22.1	25.1	0.34	2.20	12.7	
	4.5	20.7	23.6	0.38	2.38	14.0	0.46
	9	22.0	25.1	0.37	2.04	11.4	
M/PTBC8	1	16.3	18.6	0.26	1.84	12.0	0.32
	2.5	22.8	26.2	0.29	2.21	12.8	
	4.5	22.0	25.0	0.35	2.19	13.0	0.35
1 Barrer = 10^−10^ cm^3^ (STP) cm cm^–2^ cmHg^–1^ s^–1^							

**Table 4:** Selectivity of the Matrimid-based membranes, neat and MMMs (‘as prepared’, *T* = 25 °C).

Sample	Conc. (wt%)	αGas/N_2_ (-)				
		He	H_2_	O_2_	CO_2_	CH_4_
Neat Matrimid^®^	0	70.0	74.5	6.73	37.1	0.93
M/PTBC6	1	67.4	77.3	6.57	38.2	0.93
	2.5	65.9	75.0	6.56	37.8	
	4.5	54.8	62.6	6.31	37.2	1.22
	9	60.2	68.8	5.59	31.1	
M/PTBC8	1	63.4	72.4	7.13	46.7	1.24
	2.5	77.7	89.0	7.50	43.5	
	4.5	62.7	71.2	6.22	37.0	1.00

**Table 5:** Gas diffusion coefficients measured on the neat Matrimid^®^ and on the MMMs with 4.5% of fillers (‘as prepared’, *T* = 25 °C).

Sample	Conc. (wt%)	He	H_2_	N_2_	O_2_	CO_2_
		*D* (10^−8^ cm^2^/s)				
NEAT Matrimid^®^	0	676	-	0.263	1.73	0.368
M/PTBC6	1	790	147	0.361	1.80	0.430
	4.5	730	164	0.425	2.27	0.555
M/PTBC8	1	884	190	0.687	2.88	0.687
	4.5	754	-	0.282	2.24	0.505

**Table 7:** Activation energy for diffusion on the neat Matrimid^®^ and on the MMMs with 4.5% of PTBC fillers (aged samples, 3 months).

Membrane Code	Activation Energy for Diffusivity, *E*_D_ (kJ/mol)				
	H_2_	He	CO_2_	O_2_	N_2_
Neat Matrimid^®^	25.4	35.3	30.3	31.0	29.9
M/PTBC6, 4.5 wt%	19.6	17.6	29.0	30.1	34.3
M/PTBC8, 4.5 wt%	18.1	13.8	31.4	32.9	34.8

**Table 8:** Comparison among the performance of Matrimid mixed matrix membranes for CO_2_/N_2_ separation (adapted from reference [56]).

Filler	Loading (wt%)	Permeability (Barrer)		CO_2_/N_2_ Ideal Selectivity	T (°C)	Feed Pressure (bar)	Ref.
		CO_2_	N_2_				
NH_2_-UiO-66	0	8	0.28	29	25	1.4	[29]
	23	23	0.66	35			
NH_2_-UiO-67PA	23	28	0.78	36			
NH_2_-UiO-68C10	23	22	0.81	27			
NH_2_-UiO-69SA	23	19	0.63	30			
MOF-5	0	9	0.25	36	35	2	[57]
	10	11.1	0.28	39.6			
	20	13.8	0.4	34.5			
	30	20.2	0.52	38.8			
MCM-41	0	6		27	25	10	[58]
	10	7		27			
	20	8		27			
	30	10		26			
SO_3_H-MCM 41	0	6		27	25	10	[58]
	10	6		30			
	20	8		30			
	30	10		31			
Cu_MOF	0	7.33	0.24	30.5	35	2.67	[59]
	9	20.54	0.66	31.1			
	17	38.27	1.33	28.8			
	23	74.08	3.44	21.5			
	29	233.9	19.89	11.7			
	33	465	102.3	4.6			
	45	3130	1204	2.6			
MOP-18	0	7.3	0.24	30.4	35	2.67	[59]
	23	9.4	0.34	27.6			
	33	14	0.61	22.9			
	45	15.6	0.6	26			
Cu-BPY-HFS	0	7.29	0.22	33.1	35	2	[60]
	10	7.81	0.24	32.5			
	20	9.88	0.31	31.8			
	20	10.02	0.32	31.3			
	30	10.36	0.31	33.4			
	40	15.06	0.49	30.7			
ZIF-8	0	8	0.3	26.7	35	2.7	[59]
	20	9	0.3	30			
	30	13	0.53	24.5			
	40	25	1.05	23.8			
	50	4	0.18	22.2			
	60	7	0.44	15.9			
UiO-66	0	6.9	0.23	29.8	35	4	[56]
	10	7.8	0.26	29.4			
Azo-UiO-66	5	7.1	0.19	35			
	10	10	0.26	37			
	20	13	0.3	40			
PTBC6	0	9.9	0.27	37.1	25	1	This work
	1	11.3	0.30	38.2			
	2.5	12.7	0.34	37.8			
PTBC8	0	9.9	0.27	37.1	25	1	This work
	1	12	0.26	46.7			
	2.5	12.8	0.29	43.5			

## Data Availability

Data will be made available on request by corresponding author.

## Supplementary Materials

The following are available online at https://www.mdpi.com/article/10.3390/polym16040460/s1, Figure S1: Schematic diagram of the permeation testing unit, Figure S2: ^1^H-NMR spectra of Matrimid^®^ powder, PTBC8 powder and Matrimid^®^/PTBC8 membrane dissolved in CDCl_3_. The spectra were recorded on a Bruker spectrometer 400.13 MHz, at 297 K, Figure S3: DSC curves of PTBC4 (temperature range 250–350 °C) after TGA drying procedure (from 40 to 190 °C at 10 °C/min and isotherm at 190 °C for 15 min). Both heating cycles from −90 to 350 °C, cooling cycle from 350 to −90 °C, Figure S4: DSC curves overlay on second heating scan of PTBC4, PTBC6 and PTBC8. Expansion of the curves in the temperature range 250–350 °C, Figure S5: Photos of the prepared membranes, Figure S6: SEM images of samples of Matrimid^®^ and M/PTCB4 films, Figure S7: Permeability increment (*P*/*P*_0_) for CO_2_ of the MMMs compared to neat Matrimid^®^.
