# Supplementary material for: Molecularly Mixed Composite Membranes for Gas Separation Based on Macrocycles Embedded in a Polyimide
Source: Polymers (Basel). 2024 Feb 7;16(4):460. doi: 10.3390/polym16040460 (PMC10892679; doi:10.3390/polym16040460)
Supplement: Supplementary file 1 [file polymers-16-00460-s001.zip › polymers-2822218-supplementary.pdf]

Supplementary Information

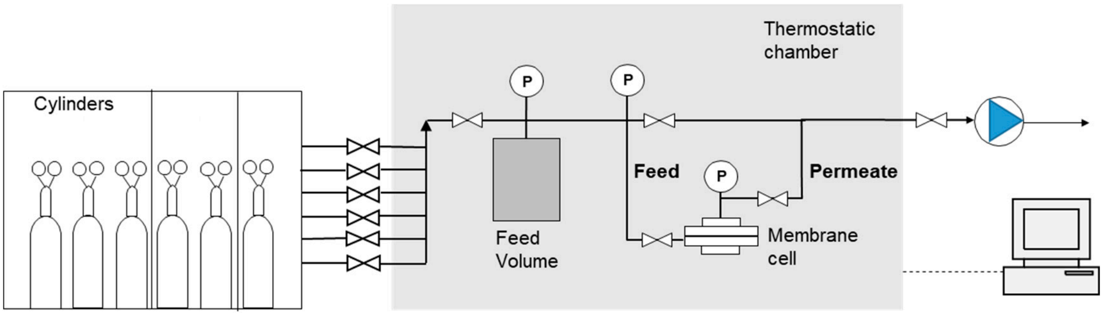

Figure S1. Schematic diagram of the permeation testing unit.

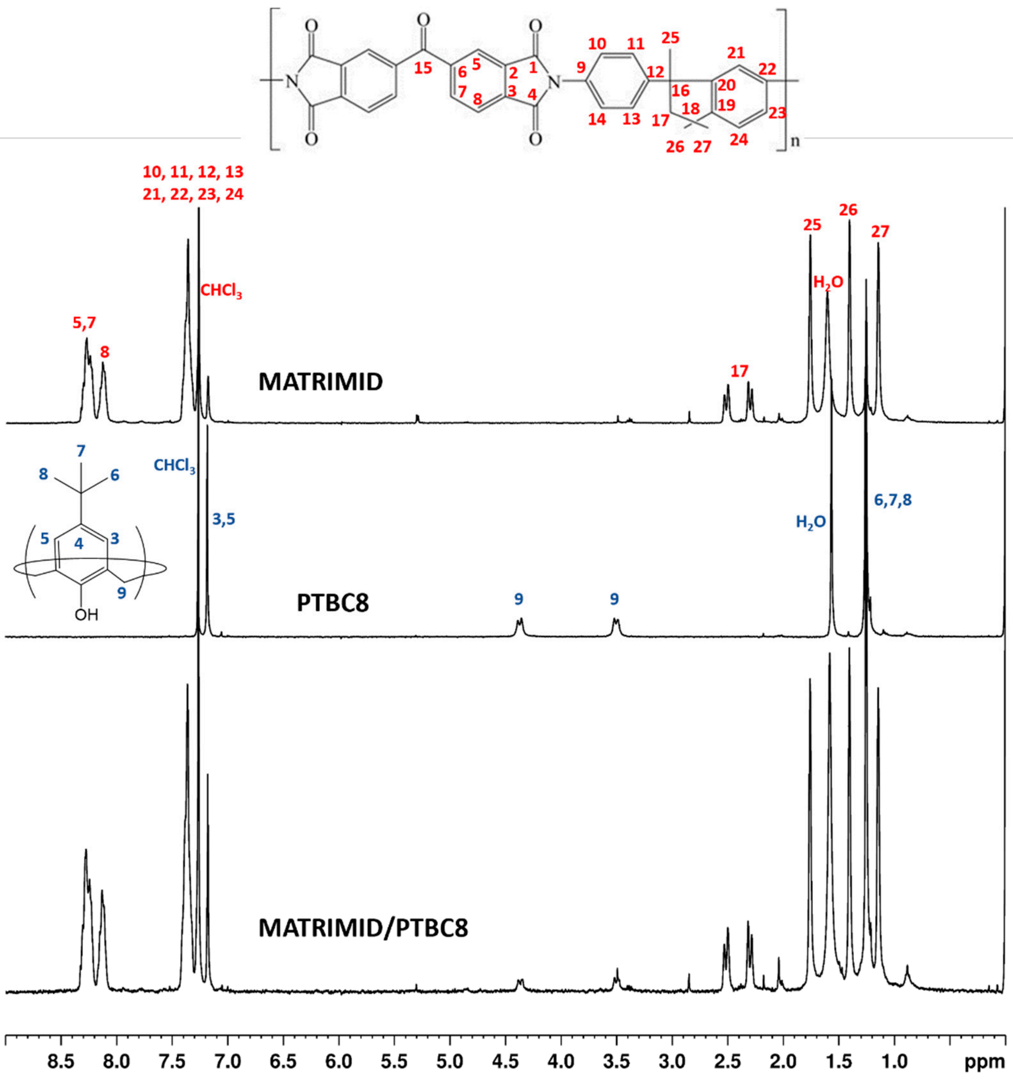

Figure S2. ^{1}H-NMR spectra of Matrimid® powder, PTBC8 powder and Matrimid®/PTBC8 membrane dissolved in $CDCl_3$. The spectra were recorded on a Bruker spectrometer 400.13 MHz, at 297 K.

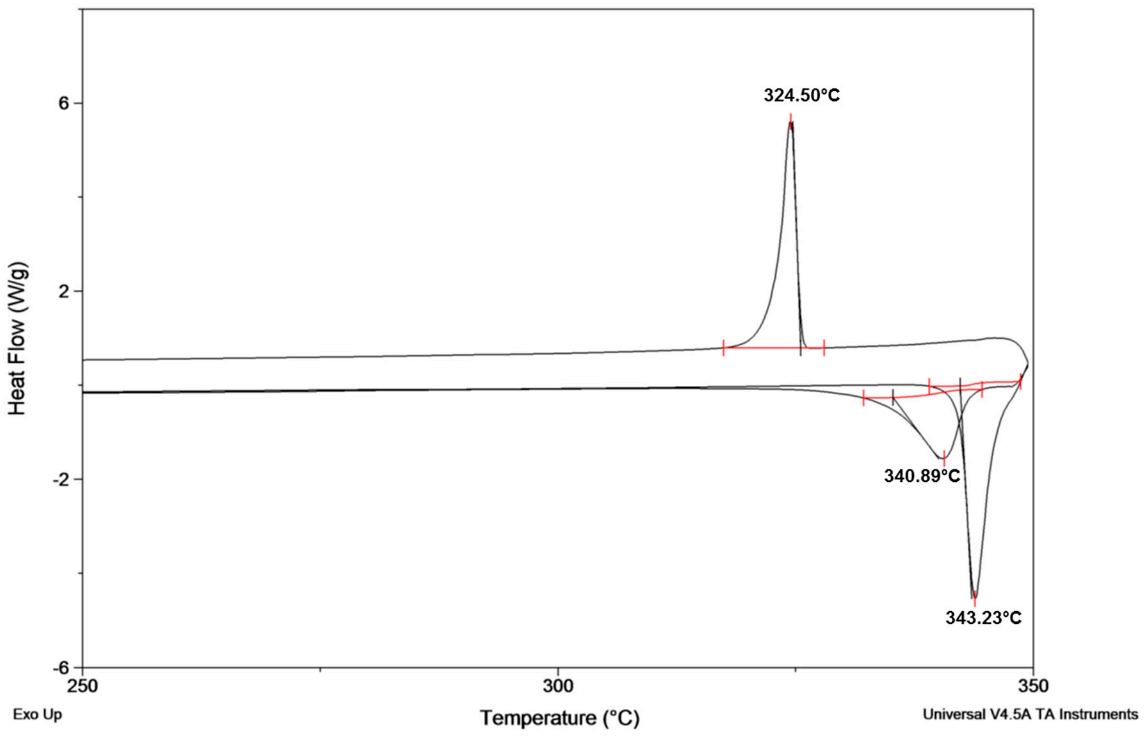

Figure S3. DSC curves of PTBC4 (temperature range 250-350 °C) after TGA drying procedure (from 40 °C to 190°C at 10 °C/min and isotherm at 190 °C for 15 min). Both heating cycles from -90°C to 350 °C, cooling cycle from 350 °C to -90 °C.

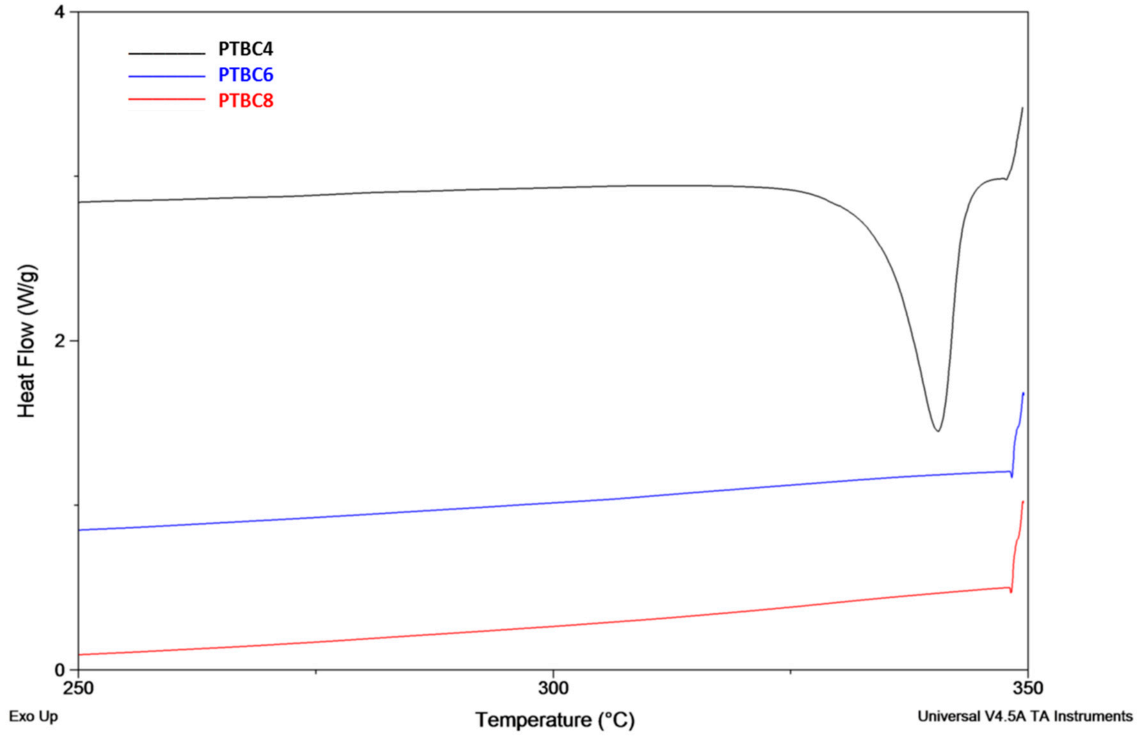

Figure S4. DSC curves overlay on second heating scan of PTBC4, PTBC6 and PTBC8. Expansion of the curves in the temperature range 250-350 °C.

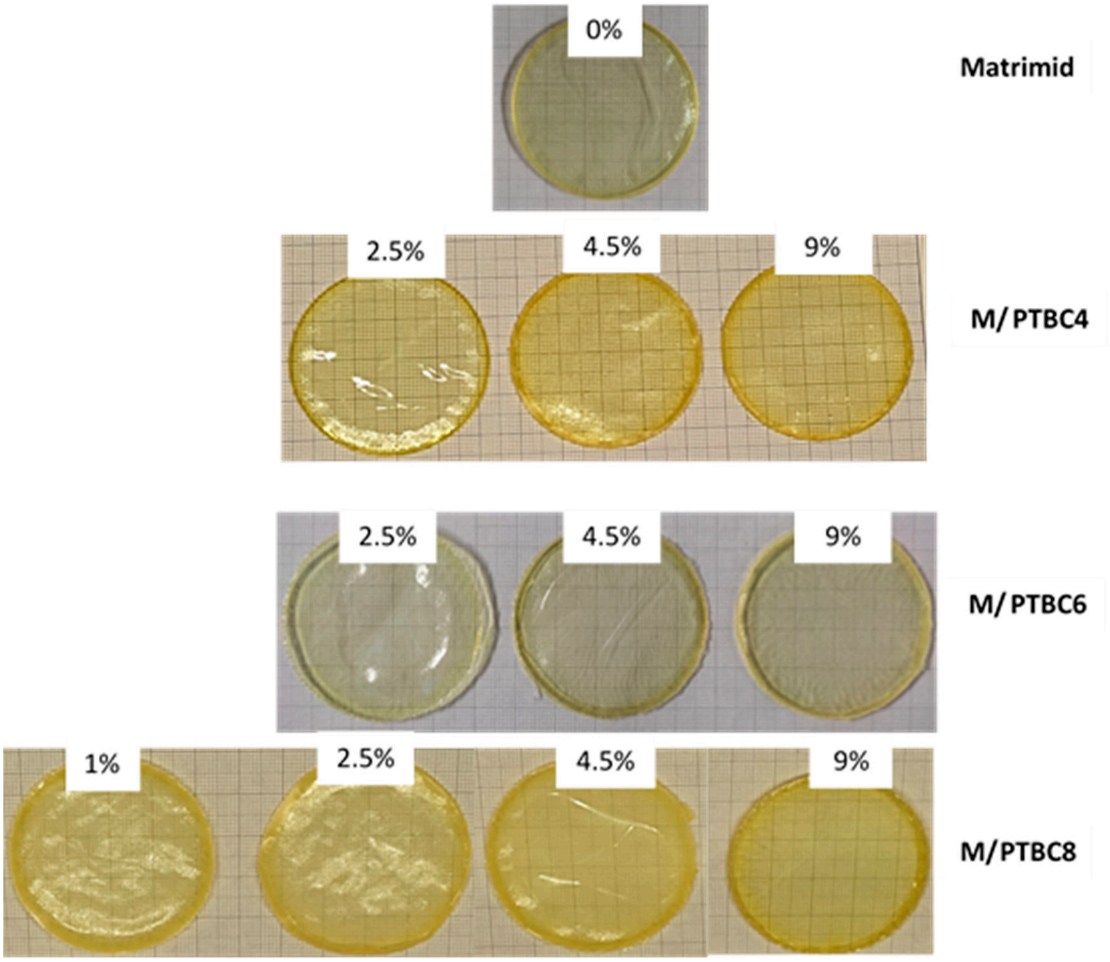

Figure S5. Photos of representative samples of the prepared membranes.

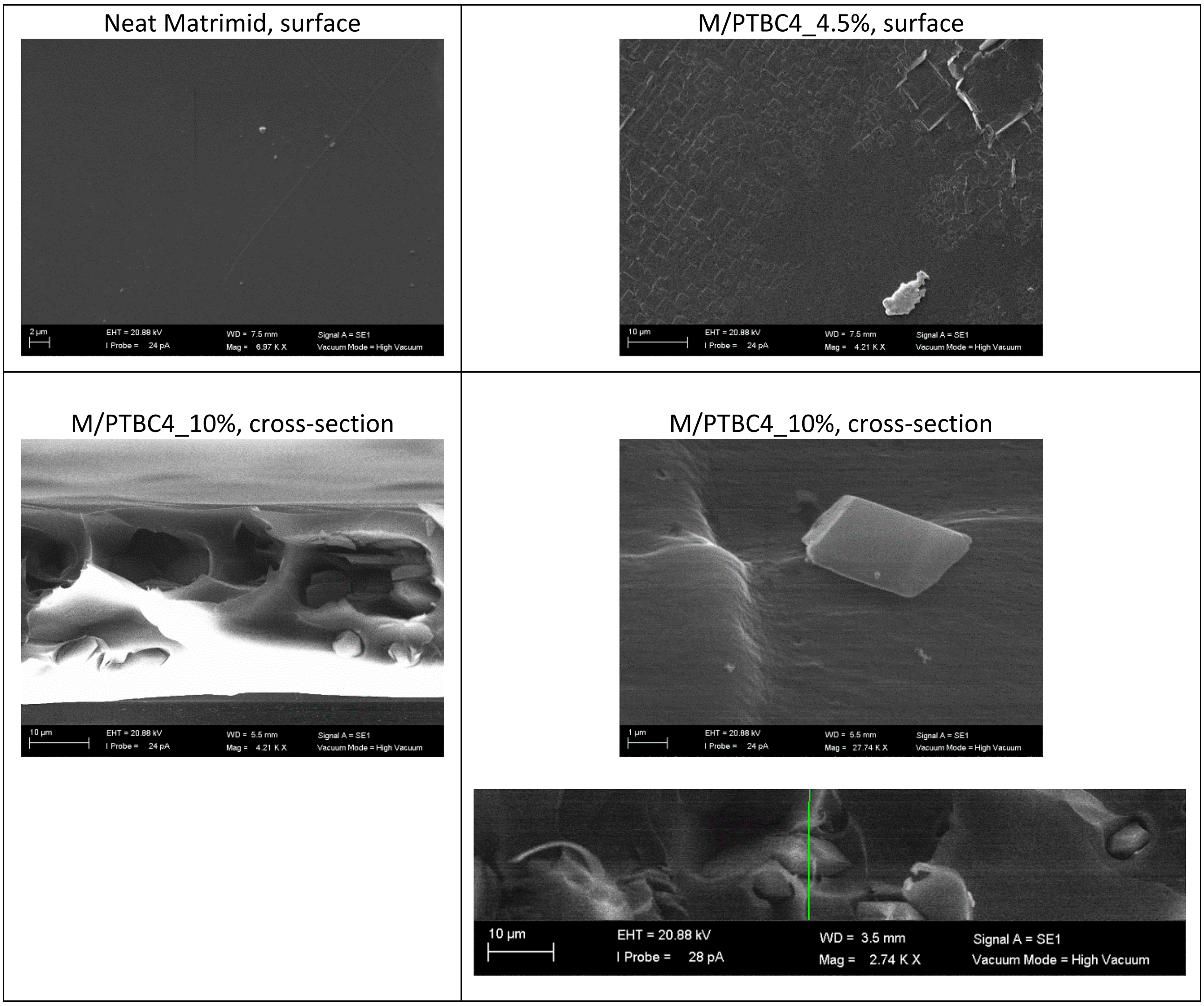

Figure S6. SEM images of samples of Matrimid® and M/PTCB4 films.

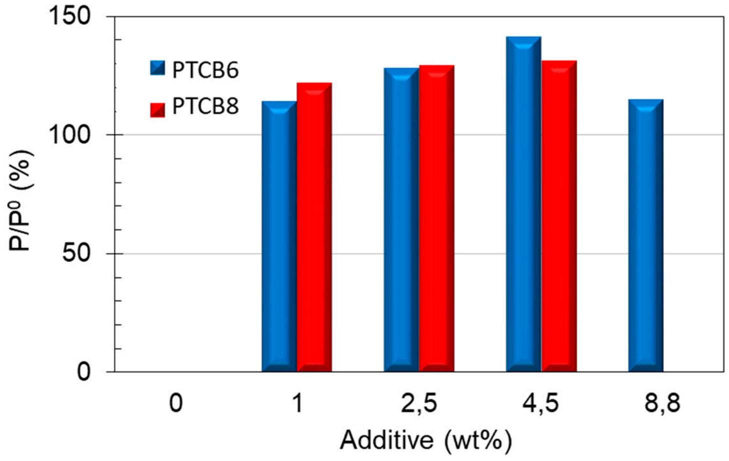

Figure S7. Permeability increment (P/P_0) for CO_2 of the MMMs compared to neat Matrimid®.
